# EMPOWERING OLDER ADULTS AND REDUCING HARM THROUGH PERSON-CENTERED ENDEAR PROGRAM

**DOI:** 10.1093/geroni/igae098.2924

**Published:** 2024-12-31

**Authors:** Julie Kim, Julie Rousseau, Jorge Sole, Sharnette Raye-Brown, Elvira Dodd, David Sanchez, Lisa Gibbs

**Affiliations:** University of California Irvine, Irvine, California, United States; University of California Irvine, Irvine, California, United States; University of California Irvine Health, Irvine, California, United States; University of California Irvine Health, Irvine, California, United States; OC Health Care Agency, Santa Ana, California, United States; OC Health Care Agency, Santa Ana, California, United States; University of California Irvine, Irvine, California, United States

## Abstract

In Orange County, California, the Office of Public Guardian (OPG) investigates whether persons referred to OPG have resources and support to manage their financial and personal affairs. In the 2021-2022 fiscal year, OPG investigated 613 referrals. The UC Irvine Center for Excellence on Elder Abuse and Neglect and OPG partnered in a quality improvement project providing a person-centered case management program, ENding and Disrupting Elder Abuse Recidivism (ENDEAR), supported by the Administration for Community Living. A case manager and referred participants collaborated to identify health and safety improvement areas that could obviate a court petition to establish conservatorship where a conservator is appointed to manage the older adult’s financial affairs, personal affairs, or both. Participants (n=20, 70% female, mean age=78) receiving case management had a mean ADL score of 1.8 (0 independence to 6 total dependence), IADL score of 6.5 (0 independence to 8 total dependence), and financial capacity score of 4.9 (0 independence to 7 total dependence). All were at risk of experiencing self-neglect. Most had access to social support (n=15), including family, friends, and neighbors but lived alone (n=16). The case manager coordinated services based on home visits, needs assessments, and participants’ communicated goals. On average, the case manager made 4.6 home visits over a mean of 123.9 days (range 18-386). Paired t-tests showed improvement in clients’ safety (t(19)=4.88, p< 0.001) and lowered risk of self-neglect (t(19)=-4.41, p< 001). Findings suggest case management services support OPG goals by protecting older adults through improved safety and reduction in self-neglect.

